# Digital Pathology: Advantages, Limitations and Emerging Perspectives

**DOI:** 10.3390/jcm9113697

**Published:** 2020-11-18

**Authors:** Stephan W. Jahn, Markus Plass, Farid Moinfar

**Affiliations:** 1 Diagnostic and Research Institute of Pathology, Medical University of Graz, Neue Stiftingtalstraße 6, 8010 Graz, Austria; markus.plass@medunigraz.at (M.P.); farid.moinfar@pathologieverbund.at (F.M.); 2 Department of Pathology, Ordensklinikum/Hospital of the Sisters of Charity, Seilerstätte 4, 4010 Linz, Austria

**Keywords:** digital pathology, machine learning, artificial intelligence, whole slide imaging, occupational health, computer vision syndrome, automation

## Abstract

Digital pathology is on the verge of becoming a mainstream option for routine diagnostics. Faster whole slide image scanning has paved the way for this development, but implementation on a large scale is challenging on technical, logistical, and financial levels. Comparative studies have published reassuring data on safety and feasibility, but implementation experiences highlight the need for training and the knowledge of pitfalls. Up to half of the pathologists are reluctant to sign out reports on only digital slides and are concerned about reporting without the tool that has represented their profession since its beginning. Guidelines by international pathology organizations aim to safeguard histology in the digital realm, from image acquisition over the setup of work-stations to long-term image archiving, but must be considered a starting point only. Cost-efficiency analyses and occupational health issues need to be addressed comprehensively. Image analysis is blended into the traditional work-flow, and the approval of artificial intelligence for routine diagnostics starts to challenge human evaluation as the gold standard. Here we discuss experiences from past digital pathology implementations, future possibilities through the addition of artificial intelligence, technical and occupational health challenges, and possible changes to the pathologist’s profession.

## 1. Introduction

Histopathology is a diagnostic discipline founded on the visual interpretation of cellular biology captured in images. The advent of digitized images to pathology has propelled this traditional field into what is now described as digital pathology (DP). Digital images and video streams can be shared in real-time, thus bridging physical distance (telepathology) between local hospitals, colleges (second-opinion), teachers and students, and between home and workplace (home-office). They can be superimposed or linked beyond what physical glass slides would permit to facilitate spatial correlation across slides and stains. Digital images lend themselves to computational pathology (CPATH), both for basic measuring and counting and for advanced machine learning (ML) tasks. Most fascinating of all, images can now be evaluated by ML for features beyond the assessment of traditional histopathology (artificial intelligence (AI)), such as to directly link images to clinical data (e.g., prognosis, mutations). New possibilities come with new challenges: extensive investments in IT-infrastructure and services, best practices for safe implementation, regulatory requirements, AI as an unaccountable “black-box”, working extensive screen times (occupational medicine), questions of cost-efficacy, and transformation of the profession by automation. This review will systematically elaborate on these topics and introduce applications already implemented or currently under investigation in DP.

## 2. From Telepathology to Whole Slide Imaging (WSI)

Initially, only single screenshots of histological images captured through a microscope’s optics were digitized mostly for detailed evaluation in research settings for documentation and teaching. The introduction of “telepathology”, a term coined in the 1980s, used a remotely operated, motorized microscope and a live view option of the microscopic slides. The setup was used for selected cases in frozen section evaluation, consultation practice, and niche applications (e.g., transplant pathology). Technical advances in scanning speed and decreased costs have made whole slide imaging (WSI) the standard for future, large-scale, high-throughput DP. Scanning of all relevant tissue is a prerequisite for a digitized histopathology work-flow to fully replace the optical microscope, i.e., for primary histopathological diagnosis. WSI requires dedicated equipment and IT-infrastructure (scanners/servers/bandwidth/work-stations) to minimize system downtime. WSI enables tissue work-up, cutting, and staining at the local pathology department, followed by immediate scanning of slides, which can be later accessed by the reporting pathologists. Pathologists can subsequently view data either on-site or remotely. Remote viewing access requires sufficient data bandwidth (typically > 10 Mbit/s) and low latency for smooth operation. This can add up to considerable IT-capacity requirements in large pathology departments. Orders for recuts, step-sections, and additional histochemical or immunohistochemical stains are electronically requested at the central laboratory and receive subsequent scanning for further online access by the reporting pathologist. Availability of glass slides upon request is necessary even in centers with long DP experience [1], as in the region of 5–10% of cases are requested for glass slide microscopy. Furthermore, the assessment of slides for polarization effects (e.g., amyloid, weddelit) cannot be performed on digital slide scans and necessitates evaluation from glass slides.

## 3. Regulatory Requirements for WSI for Patient Diagnostics in Europe and the US

At present, WSI scanners are cleared to be used in the European Union under directive 98/79/EC of the European Commission for in vitro diagnostic (in vitro diagnostic medical device directive (IVDD)) [2]. DP software, as standalone software, such as WSI viewers or automated image analysis for specific tasks (e.g., immunohistochemical quantification), can also receive the CE mark IVD-MD (medical devices). Essentially, conformity is based on a self-declaration of the manufacturer. Scanners and associated software of numerous manufacturers are currently CE-IVD labeled such as those of Philips, Roche/Ventana, Leica/Aperio, Hamamatsu, 3DHISTECH. Under the new in vitro diagnostic medical device regulation (IVDR) of the European Parliament, all in-vitro medical devices, including slide scanners and digital pathology software, are to apply for CE-marking as of May 2022. The IVDR will require a performance evaluation with a scientific validity report and an analytical and clinical performance report. Thus, European approvals in DP will more closely resemble the current Food and Drug Administration (FDA)-approvals for the US-market [3]. Only two WSI platforms have so far received FDA approval for primary surgical pathology (histopathological diagnosis) in the US. The first approval was granted in 2017 to the Philips IntelliSite Digital Pathology Solution. It is a closed system that comprises a scanner/image management system and display. Approval was based on a non-inferiority study [4] of close to 2000 slides of different histopathological entities. The approval does not extend to frozen sections, cytology, or non-formalin fixed paraffin embedded (FFPE) hematopathology specimens. As of September 2020, the second platform to have been granted an FDA approval for primary diagnosis is the DP Module by the Swedish company Sectra in conjunction with Leica Biosystem’s Scanner AT2 DX, itself FDA approved in May 2020.

## 4. Concordance of Digital Pathology (DP) with Glass Slides

Multiple studies have compared WSI glass slides to digital slides and evaluated their concordance. In the aforementioned non-inferiority study used for FDA market authorization [4], an equal discordance rate between glass slides (4.6%) and WSI (4.9%) slides was seen when each slide type was reevaluated ≥4 weeks after initial diagnosis. Major WSI vs. glass discordance rate was not significant at 0.4% (95% confidence interval (CI) (−0.3–1.01)). In a review of discordant cases, no consistent inaccuracies due to WSI were observed. Other studies have found concordance rates to range from above 90% [5] to over 95% [4,6,7]. In the recent 2020 meta-analysis by Azam et al. [7] comprising 25 publications and 10,410 samples, a 98.3% (95%CI (97.4–98.9)) concordance rate was described. The majority of discordances (57%) were related to the assessment of nuclear atypia, grading of dysplasia and malignancy, followed by challenging diagnoses (26%, e.g., focally invasive/malignant lesion) and identification of small objects (16%, e.g., *Helicobacter pylori*, mitoses). Generally, the most frequently and consistently mentioned problems in routine DP refer to detecting microorganisms (*Helicobacter pylori*), as well as recognizing mitoses and nuclear features in dysplasia.

Professional organizations have issued guidelines for DP use including, but not limited to, technical specifications, such as among others the College of American Pathologists (CAP) [8], the Digital Pathology Association [9], the European Union [10], the Canadian Association of Pathologists [11], the Royal College of Pathologists (RCPath) [12,13] and the Bundesverband Deutscher Pathologen (Professsional Association of German Pathologists) [14].

## 5. Critical Quality Parameters in WSI for Diagnostic Imaging

If the manufacturer has not performed external validation, in-house validation should be performed on a sufficient number of representative cases, including evaluating auxiliary techniques (immunohistochemistry, special stains). The College of American Pathologists (CAP) guideline statements [8] recommend a minimum of 60 hematoxylin eosin (HE)-slides and at least an extra 20 slides for each auxiliary technique. The time between digital and glass slide comparison should be at least two weeks to guarantee unbiased evaluation (“washout period”) by the same person. A summary of relevant points for DP accreditation according to ISO 15189 is highlighted in [15].

Perceived minimum requirements for dedicated DP hardware differ between individual pathologists, institutions, and the use-case, i.e., whether DP is used for primary diagnosis or only an adjunct to optical microscopes. Office grade screens and computer hardware operated by a computer mouse are widely available. However, high-definition monitors dedicated to DP or even special digital controllers akin to classical stage handles of a microscope are less frequently used but increase ergonomics and can be expected to further acceptance of DP by pathologists.

Accurate color rendition is essential and necessitates WSI scanners’ regular calibration against a standardized reference (established: “IT8.7/1 target”, 28 greys/264 colors). Color deviations are corrected through software profiles (ICC, International Color Consortium, profiles) for each scanner. Monitors are also advised to be color-calibrated, taking into account the need for recalibration due to different lighting conditions, which can be conveniently achieved by self-calibrating monitors. Interestingly, a study [16] found equal diagnostic accuracy but faster diagnoses on color-calibrated than uncalibrated images. In this vein, Norgan et al. [17] demonstrated a high agreement (κ > 0.75) between calibrated and uncalibrated monitors for 90% of pathologists tasked with counting mitoses and searching for *Helicobacter pylori*. Regarding screen size and resolution 27-inch WQXGA and 32-inch UHD screens have been recommended for diagnostic DP by Haroske et al. [14]. Higher screen resolution was found to decrease time to diagnosis [18], but conventional optical microscopy was the fastest still, in line with another study [19] reporting increased time to diagnosis through DP. Abel et al. provide a recent overview of the selection of displays for DP [20].

Usually, WSI captures pre-scans of the whole slide at low resolution, and only tissue detected on the pre-scan is then scanned at high-magnification. Therefore, tiny tissue particles might elude automatic inclusion for final scanning and would be lost to evaluation. The Professional Association of German Pathologists [14] recommends adjusting settings to include all tissue clusters beyond 3 × 3 cells. It encourages the implementation of an option for the pathologist to compare preview images to final high-resolution scans to avoid missing small particles. The problem potentially extends to slide areas not coverslipped but holding material of possible diagnostic relevance. In their routine implementation, Stathonikos et al. [21] used the human control by a technician immediately after scanning to verify complete WSI and to initiate rescans for slides with out-of-focus areas (see below) before delivery to the pathologist.

In conventional microscopy, pathologists regularly evaluate multiple focus planes. WSI scanners per default acquire only a single focal plane. Some scanners can technically acquire multiple planes, referred to as “z-stacking”, but acquisition times and data increase proportionally (e.g., fivefold for five focal planes), and z-stacking has mostly been restricted to research. Hence, for routine diagnostics, tissue might be out of focus if not in the one scanned plane (e.g., at folds), possibly necessitating rescans. Evaluation of a single focus plane only can contribute to difficulties in identifying microorganisms (HP), mitoses, and dysplasia. These are known challenges in DP, and settings in which pathologists commonly use multiple focus levels on optical microscopes. Furthermore, blurred lines on scans corresponding to borders of digitally merged single images, so-called “stitching artifacts”, can appear on WSI images and obscure relevant details. Out of focus areas can be addressed by manually or automatically defining additional focus points. If WSI remains unsatisfactory, the case must be deferred to glass-slide evaluation.

Data storage of diagnostically used WSI data on consumer hard drives is inadequate, and data storage on professionally maintained and secured servers is mandated. Developed for radiology, these systems are referred to as PACS (Picture Archiving and Communication System). Stored data should produce images identical to the time of reporting to guarantee that subsequent, possible medico-legal evaluations are not biased by reviewing image data with divergent image quality [14]. DP data can be stored in open or proprietary formats. The latter can lead to incompatibility between scanners or to data archives that have to be converted when scanners with new file formats are procured. In addition, extramural expert consultation requires the compatibility of file formats. Therefore, the use of the open DICOM (Digital Imaging and Communications in Medicine) standard is encouraged.

In conclusion, the state of the art technical implementation of WSI in routine diagnostics is complex, requires expertise, human resources, and a continuous focus on technical advances. Lastly, pathologists may need external professionals to define specifications for major hardware procurements and compare vendors’ offers in-depth meaningfully [21].

## 6. The Integrated DP Work-Flow

Once scanned, cases are assigned to a reporting pathologist. DP facilitates dynamic workload balancing or reallocation of cases in the event of, e.g., sick leave. Regarding digital slide evaluation, studies on image perception have shown that pathologists first evaluate the digital image by an initial glance in order to assess the overall image properties, such as spatial distribution, texture, size, and color, and then quickly focus on regions of interest (e.g., a suspected carcinoma focus). Randell et al. [18] reported that higher resolution monitors are advantageous for faster identifying these areas on low-power digital images, which are subsequently evaluated at high-power. Tools using AI can highlight such regions of interest [22] for IT-assisted screening. Applications for magnification-sensitive tracking of digital slide movements can help to prevent missing small particles [23]. Tools for measuring and quantification, launched directly from the viewer, allow for instant documentation of results. Multiple slides can be displayed side-by-side and interlinked to be moved synchronously or superimposed, facilitating spatial correlation of different stains. Further auxiliary, mostly AI-powered applications, have either already been integrated into commercially available platforms or have been published to be suitable for such integration. Use cases include immunohistochemical Ki-67 evaluation to determine a tumor’s proliferation rate [23], challenging to quantify biomarkers (e.g., immunohistochemical PD-L1 staining [24,25]), evaluation of residual cancer burden after chemotherapy [26], or cancer detection and classification algorithms (e.g., in prostate cancer [27,28,29]). In a further step towards automation, reports may then be composed using AI-based speech-recognition.

An early example of a routine AI-application embedded in the work-flow after report generation but before actual sign-out is the second-read Galen^TM^ system (Ibex Medical Analytics, Tel Aviv, Israel) on the Philips IntelliSite platform [28] (for details see the chapter on CPATH). The software can be configured to analyze the ready histopathology report as well as images of prostate core needle biopsies in order to automatically alert the pathologist to human vs. AI discrepancies before final sign out. AI-for cancer prescreening has also been proposed to triage cases for reporting and to optimize the workload distribution. In the future, automated prescreening may even replace human evaluation [30] in cases negative for cancer.

**Figure 1:**
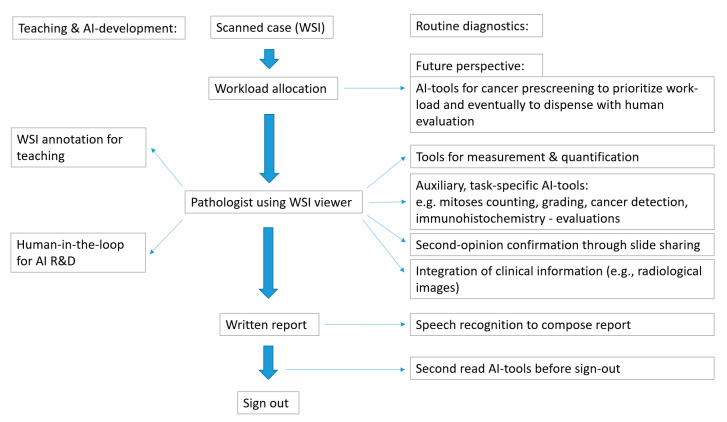
Outline for an integrated digital pathology (DP) work-flow.

Human feedback useful for AI-development can also be integrated in the DP work-flow. AI applications have been shown to benefit from human input for increased classification performance and reduced numbers of digital slides required for training, a concept termed “Human-in-the loop” [31]. Lutnick et al. [32] have recently demonstrated the use of a routine WSI viewer (Aperio ImageScope viewer) for a human-in-the-loop interaction with a convolutional neural network (DeepLab v2). A small number of WSIs were initially annotated by a human and subsequently classified by the algorithm. The algorithm was trained through repeated rounds of correction by human feedback and ever more accurate AI-classifications. This resulted in a considerable efficiency improvement in training the algorithm, so that only a small number of initially annotated WSI (<5 annotated images) were needed. The algorithm was then able to identify intact and atrophic renal glomeruli and areas of tubular atrophy in renal biopsies. Thus, human interaction could be incorporated into the training of the AI-algorithm through graphic annotations on a routine DP interface. Figure 1 depicts an outline for an integrated DP work-flow.

## 7. Experience from DP Implementations in Routine Diagnostics Using WSI

Comparatively few reports based on digital pathology experiences to fully replace optic microscopes for routine diagnostic purposes are available. The Eastern Quebec Telepathology Network (EQTN) is one of the most extensive real-life applications for DP aimed at providing a DP histopathology service to the widely dispersed population in Canada [33,34,35]. Taking up service in 2011, the network includes 22 hospitals with a catchment area of 1.7 million people. An EQTN evaluation project has published the implementation results [34,36]. The network provided remote intra-operative consultation and histopathological second opinion. It was initiated due to a lack of pathologists in remote rural areas, which endangered state-of-the-art surgical service provision. A 2018 evaluation [33] confirmed the reduction of two-stage surgeries and patient transfers to urban centers. Service-breaks and diagnostic delays decreased upon the introduction of DP. Telepathology did not help recruit and retain pathologists in remote areas as pathologists wished to work in a team. In locations without on-site pathologists, duties, remuneration, and legal responsibilities were transferred from the pathologists to technical assistants, altering the department’s internal structure. Generally, the EQTN experiences seem valuable for its long duration and for raising issues beyond mere technical aspects commonly described in more limited, academic studies.

Experiences from a long-running DP implementation in Sweden since 2006, using WSI at two sites (Kalmar County Hospital and Linköping University Hospital) were reported by Thorstenson et al. [37] in 2013, based on 500,000 slides at time of publication, with now around 1.8 million slides scanned to date [38]. In a parallel implementation of DP and glass-slides, the pathologists were free to choose between digital and glass slides. Overall, 38% of cases were diagnosed digitally, but pathologists reported frequent switching between digital and glass slides, including within the same case, highlighting perceived shortcomings for pathologists at the time to confidently sign-out reports with DP.

In 2017, Evans et al. [39] published their five-year DP implementation experience on 6700 cases/35,500 slides and reported a >90% digitally only sign out rate. Conversely, almost 10% of cases required additional glass slide evaluation. Hanna et al. [1] in 2019 also reported on the importance of glass slide availability on request in DP employed for primary diagnoses. The authors published their DP use at the Memorial Sloan Kettering Cancer Center, US, including a DP experience survey for pathologists’ feedback; 48% of respondents would not have felt comfortable with DP sign-out without glass slides available, and 15% felt uncomfortable even with glass slides available. Nevertheless, 91% thought DP reduced turnaround times, helped to decide on repeat ancillary studies (96%), and was useful for prior case review (83%).

A UK teaching hospital has been using DP for the primary diagnosis of all histology slides since September 2018 and published their experience in 2020 [40]. The authors laid out technical validation and suggestions for training DP newcomers. They comprehensively listed possible areas of difficulty and pitfalls to avoid in DP evaluation according to individual fields of histopathology (e.g., gastrointestinal, breast, hepatobiliary etc.). Their report adds eosinophils, neutrophils, mast cells, amyloid, weddellite, and mucin to the list of difficult to evaluate features. Lastly, the authors described difficulties for pathologists to apply the commonly used, systematic meandering of glass slides for complete slide evaluation to digital slides. They advise actively training DP newcomers on substitute WSI-optimized techniques to guarantee complete visual evaluation of the digital slide.

Furthermore, a UK pathology laboratory focus group published their views in 2019 [41] on a set of open questions on the imminent transition to DP at their department (Oxford University Hospitals, NHS Trust). They identified benefits (e.g., collaboration, teaching, cost-savings) but also barriers to transition (e.g., standardization and validation, training, cost-effectiveness, data storage) and proposed the setting up of multiple pilot centers in the UK. These early adopters should then create national guidelines and provide cost-effectiveness analyses, possibly with the help of a project manager to define appropriate variables and oversee baseline measurements of the cost analyses.

A recent publication [23] on the experiences of the successful DP implementation at the Dutch University Medical Center in Utrecht focuses, among others, on the technical challenges and solutions since the initiation of DP in 2007. The authors describe their experiences and the pitfalls regarding laboratory management system integration and the work-flow optimization over two successive scanner generations. The first generation of scanners was in use for seven years, slightly longer than the five-year life span generally assumed for heavily used routine DP scanners [42,43]. Furthermore, data storage considerations for their large (2 petabyte) digital archive and the benefits of the recent integration of image analysis tools are addressed. Apart from DP implementation experiences published in the academic literature, a wealth of information is also available on commercial websites, especially of manufacturers of DP scanners.

Undeniably, the current COVID-19 pandemic and the associated social distancing restrictions have been an unexpected boost to DP. A UK guidance study published by the RCPath [13] provides recommendations on the use of DP in the context of the COVID-19 pandemic. A recent study [44] has looked at “emergency” use of DP in the US during the COVID-19 pandemic on home-office work-stations. The study was possible due to the temporarily waived regulatory requirements on DP workstations during the pandemic. The study reported nearly complete glass-to digital concordance from a home-office regarding diagnosis and parameters of clinical relevance. Participants reported working on non-DP-dedicated hardware as feasible, even though 42% of study participants worked on a below 14 inch screen. Available home internet connection speeds were deemed sufficient (range 3–385 Mbit/s) with 13% using a <20 mbit/s connection. WSI latency was unrelated to computer hardware performance but dependent on internet connection speeds. Ninety percent of pathologists reported being “comfortable” or “very comfortable” (top two items on a five point Likert-scale) with DP and the option of glass slides available on request.

## 8. Medical Education and the Consultation Setting—Advantages and Challenges

Medical education might be the most widely used application of DP to date, and its use is relatively straightforward. Dedicated digital microscopes for presentation can visualize the slide reviewing process by live video streaming to give insight into the histopathological evaluation strategy for training purposes. Digital images can be included in presentations, annotated, used for exams, and made available for self-and remote-study [45]. DP in teaching is for illustrative purposes only and free from medico-legal calamities. Therefore, most issues that beset the use of DP for primary histopathological evaluation, such as exact color rendition, resolution, completeness of scans, out of focus areas, long-term data storage, etc. are of secondary importance. Therefore, medical teaching is often the area of the first contact between pathologists and DP. 

Consultation pathology is regularly invoked as an ideal application for DP. Nevertheless, HE slides together with FFPE blocks are often sent for second-opinion as cases frequently need to be recut for additional immunohistochemical stains or molecular analyses. Moreover, consulted experts might prefer slides to be stained in their laboratory since familiar, standardized work-up can be of value in challenging cases. Image quality and proper calibration of digital slides, as well as technical compatibility between DP systems, are further issues. Given these limitations, some experts in consultation pathology are currently reluctant to base binding decisions in challenging cases on digital images only, not least for medico-legal reasons. From a technical perspective, digital consultation pathology necessitates a vendor-independent platform for slide review of different provenance. A Dutch initiative has recently reported the setting up of a platform for the exchange of WSI for teleconsultation, and virtual expert panels [46].

## 9. Computational Pathology (CPATH)

WSI is an ideal launchpad for computational pathology (CPATH). CPATH is a blanket term for an array of methods that aim to add diagnostic value by IT-assisted analysis of the image data. It spans a wide range of applications that comprise in order of ascending complexity image-enhancing features, measuring and quantification, graphical highlighting/pre-selection by heat mapping, and lastly, fully automated assessments. A recent white paper [9] from the Digital Pathology Association (DPA) provides an overview of definitions, best practices, and regulatory guidance recommendations.

The vast majority of current applications refer to image analysis to improve the accuracy and reproducibility of morphological variables conventionally assessed by the pathologist through visual estimation or manual counting. Such tasks include measuring distance to resection margins, size of lymph node metastases in breast cancer, or depth of invasion used for staging and prognostication (e.g., melanoma).

Examples of evaluation by traditional image analysis and now ML in DP include quantification of mitotic index [47], proliferative index by Ki-67 immunohistochemistry [48], nuclear estrogen- and progesterone receptor positivity in breast cancer, as well as immunohistochemical HER2 staining [49]. These applications have variably been included in DP viewers for easy operation. Also, an application to estimate residual tumor cell content after chemotherapy has been developed [26]. A more complex application for routine diagnostics has been made available as a CE-IVD approved software for the Philips IntelliSite Pathology Solution in 2018. The software quantifies intratumoral and invasive margin T-cell infiltration in colon cancer (Immunoscore) [50] in order to predict the risk of recurrence at five years in stage II and III colon cancer [25]. For a recent overview of deep-learning AI-applications and their performance characteristics see [51]. As long as CPATH is used to evaluate parameters also amenable to human evaluation, the pathologist, regardless of the underlying technique employed, can check results for plausibility, and final accountability rests with the pathologist.

AI clearly creates most of the excitement around DP. The term is commonly known to the broader public and is somewhat vaguely defined at the technical level: It denotes applications that use ML as a technical basis to execute tasks that are generally assumed to be reserved for human, intelligent behavior due to their complexity. “Machine learning” and “AI” are commonly used synonymously, although ML is a subgroup of AI applications. ML can broadly be divided into supervised and unsupervised ML. Both forms need large datasets to train the ML algorithm. With supervised ML, human-made classifications (e.g., features/histotypes in pathology) are used to train the algorithm to classify new cases. Unsupervised ML does not need user-specified criteria, but the algorithm establishes groups of cases with similar image properties on its own. These patterns can coincide with established morphological classifiers but may also be unbeknown to the pathologist. For safe clinical use, ML training sets have to be large enough to ideally/theoretically cover the whole spectrum of diagnostic possibilities. This necessitates extensive datasets, especially in unsupervised ML, where the nature of the relevant features that need to be covered are not known.

The performance characteristics of ML/AI algorithms have to be rigorously evaluated for their diagnostic reliability. How the trained algorithm derives at a conclusion is generally unknown. This phenomenon is commonly referred to as AI being a “black box”. Efforts are made to render the AI algorithms more transparent and allow for human scrutiny to detect undesirable “behavior” in AI. Increased transparency [52] through “explainable” AI (XAI) aims to avoid hidden biases in AI algorithms and tries to convert AI from a “black box” into what has been coined a “glass box” [53,54]. Concepts include visualization of pixels that impacted decision-making and the presentation of the training image that most influenced the decision [52]. Conversely, AI has sparked renewed interest in the pathologists’ evaluation strategies to improve ML [55]. The human-in-the loop approach to improve training performance in ML has already been mentioned in conjunction with the description of the integrated DP work-flow.

More than 60 applications in ML/AI have already been approved by the FDA in the US [56] for radiology, cardiology, and internal medicine/general practice so far, but none for pathology. AI has recently been employed with great success to prostate cancer detection and classification [27,29,30]. The Galen^TM^ Prostate software (Ibex Medical Analytics, Tel Aviv, Israel) claims to be the first AI-based pathology system embedded into routine pathology practice [28]. It has been CE-IVD marked in Europe and commercially launched this year. The algorithm detects prostate cancer in whole slide images of core needle biopsies and is designed as a second-read system to avoid missed diagnoses. The prostate cancer is assigned to either a low-grade group comprising Gleason-Score 6 and atypical small acinar glands or a high-grade group corresponding to Gleason-Score 7–10. The presence and the extent of Gleason-pattern 5 are reported, as is the finding of perineural invasion and the percentage of cancer per biopsy core. In an external validation set, the specificity for cancer detection was 97.3% (95%CI (94.4–98.7)) at a sensitivity of 98.5% (95%CI (94.1–99.6)). The software was implemented for routine practice at a large Israeli pathology department and performed a second-read evaluation on more than 900 cases before final sign-out. The algorithm led to 560 cancer alerts, of which 9% were followed up by additional levels or immunohistochemical stains. This additional work-up up led to one case being detected by the algorithm but missed by the pathologist.

CAPTH has also successfully been applied for research purposes to link digital images to clinical data directly. ML/AI has demonstrated the ability to infer the presence of mutations in tumor tissue from a tumor’s WSI data, thus extending histomorphological evaluation beyond what is amenable to a human interpretation. ML using so-called “deep learning” algorithms predicted the 10 most commonly mutated genes in non-small cell lung cancer [57], mutations in melanoma [58], and prostate cancer [59]. Furthermore, WSI images can also be linked to prognostic clinical data by ML/AI, as demonstrated for the successful prediction of patient outcome in colon cancer from image data [60,61].

An AI-based application meant to aid in the correct classification of neoplasias is Page.AI. Still in development, it was granted FDA breakthrough-designation in early 2019 [62]. It is a software that uses AI in order to match unknown tumor morphologies to images of known diagnoses in the database. Under a license agreement with the Memorial Sloan Kettering Cancer Center, US, around 1 million slides are available to the project, with another 4 million slides intended for scanning.

For all their impressive results, ML/AI can be negatively affected by a range of common factors, including blur, tissue folds, tissue tears, and color variations [63]. Algorithms for color normalization and color augmentation have been developed. Detection of novel objects can be problematic for AI algorithms, which may forcibly classify foreign bodies or rare cases not included in the training datasets into predefined groups. Attempts at solving this problem of “outlier detections” in histopathology are still in their early stage [64].

Numerous so-called “challenges” have demonstrated the advances of ML/AI in the field. Examples include the Grand Challenge on BreAst Cancer Histology (BACH) [65], and the Cancer Metastases in Lymph Nodes Challenge [66]. These competitions are hosted in the form of image datasets put out to the public. Participants are free to download the data and apply their algorithms. A winning algorithm is determined, ranked according to e.g., accuracy and computing time required. Challenges have been announced for DP, among others in breast-, prostate-, lung- and neuropathology. It is from an early mitosis-detection competition in 2012 [67] that an ML-based deep learning algorithm took the lead and highlighted the superiority of ML over non-ML approaches for advanced CPATH.

## 10. Cost-Effectiveness Considerations

Efficiency gains are frequently invoked to advocate DP implementation. If DP use is limited as for biomarker evaluation/quantification or second read tasks, total costs will increase as DP costs are incurred in addition to glass slide evaluation. Cost-effectiveness can thus only eventually be achieved in a completely digital pathology work-flow (primary digital diagnosis), but which in turn still necessitates the availability of glass slides on request. To our knowledge, no real-life cost-efficiency analysis for full DP implementation with sufficient (>5 years) follow-up has been reported. In 2019 a publication described the DP implementation [1] at the Memorial Sloan Kettering Cancer Center’s pathology department in the US. This academic center produced >125,000 slides a month as of 2017. The transition from research to routine WSI was initiated in 2015 and implemented on a subset of cases. The authors reported gradually increasing WSI up to around 23,000 digital slides per month in mid-2017. DP implementation led to a 93% reduction of glass slide requests. Ancillary immunohistochemistries decreased from 52% before DP implementation in 2014 to 21% in 2017. Yearly immunohistochemistry savings of 114,000 dollars were anticipated, and overall cost savings through DP were estimated at 267,000 dollars per year. Accounting for clinical WSI setup and maintenance costs, the breakeven was estimated for 2021, seven years after initial clinical DP implementation. Apart from immunohistochemistry, cost savings were due to reduced labor costs and vendor services, including cost savings from decreased glass slide transport. 

Another estimate on the cost efficiency of DP for primary diagnosis was published in 2017 [43], taking the author’s own UK teaching hospital (80,000 specimens, 45 full-time equivalent consultants, 9 million pounds annual departmental budget) as a model. Of note, the authors assumed that all efficiency gains could be financially recouped, which realistically will seldom be the case (e.g., due to reimbursement schemes and limited flexibility of work contracts). According to their estimates, DP costs would break even after two years at an overall 10% productivity improvement and after one year at 15%. At only a 5% productivity improvement, DP would amount to a permanent financial loss compared to glass slide pathology. The cost-efficiency analysis by Ho et al. [68] has also included more widely evaluated cost-savings through DP due to presumed avoidance of over- and undertreatment costs as well as from laboratory consolidation/efficiency gains that could be achieved in the distributed health network of the Pittsburgh Medical Center.

In summary, cost-efficiency analyses to date are few, often estimated and derived from academic single-center experiences that cannot easily be generalized. Notably, even if costs to the healthcare system are diminished, these savings generally do not translate into costs saved for the pathology department.

## 11. Digital Pathology and Occupational Health—Computer Vision Syndrome (CVS)

Occupational health aspects are rarely mentioned in DP implementation reports at all, and if so, take a low priority. We are not aware of a systematic evaluation of occupational health issues in DP to date. Conversely, health complaints do not feature prominently in unstructured DP feedback, but comparatively unspecific requests for optimized display configuration and input devices are frequent. Whether this pertains to requests for more time-efficient hardware setup or includes unspecified health concerns remains unclear. CVS or digital eye strain is defined as the combination of eye and vision problems associated with the use of electronic screens. Between 64% and 90% of computer users experience visual symptoms such as dry eyes, headaches, eyestrain, burning eyes, diplopia, and blurred vision when using (computer) screens [69]. Rossignal et al. [70] have reported increased CVS incidence in individuals looking at screens over four hours a day. CVS can lead to increased work breaks, and its economic impact is significant [71]. Similar to optical microscopy, the problem also extends to musculoskeletal injuries, and these have also been included as extraocular manifestations of CVS by some [72]. Musculoskeletal injury from computer use has been estimated to account for at least half of work-related injuries in the US [73].

Contrary to optical microscopy, which projects its image at infinity, computer screens require constant near focusing and have shown to reduce the eye’s blinking frequency. This, in turn, diminishes the ocular film and eventually leads to dry eyes. Concerning digital screens, an uncorrected especially astigmatic-refractive error, the angle of view towards the screen, medications, as well as glare, contrast, and font size are all understood to contribute to CVS.

Experiences from a Dutch DP routine implementation [21] reported on 23 pathologists, of whom 65% regarded DP to be “ergonomic” or “very ergonomic” (top two items on a four-item Likert scale), but close to 10% experienced DP as only “slightly ergonomic” (lowest item). In addition, the study descriptively evaluated musculoskeletal injury through self-reporting, but evaluation of ocular discomfort was missing. Head/neck and shoulder complaints were reduced by approximately a third with DP, but wrist/lower back/and feet complaints were of equal frequency and wrist complaints slightly elevated, possibly due to extended computer mouse usage in DP. A 10% rate of DP users discontent with DP ergonomics was also reported in [37], but complaints might be higher in close to full-time DP reporting, as only a third of pathologists opted to sign out more than half of their cases digitally. In summary, more work is urgently needed to address CVS and musculoskeletal complaints in DP, before DP is widely introduced for primary histodiagnoses.

## 12. Digital Pathology and the Pathologist’s Profession

Digitization has reshaped employment, professions and fueled globalization. If indeed DP becomes transformative, it would be naive not to assume it will reshape the pathologist’s job market. To what extent AI can replace the human pathologist waits to be seen. A 2016 study by McKinsey [74] assessed the current automation potential across US professions and found the lowest potential with professions “managing others” at 9% of work-time, together with those applying expertise at 18%, the latter applicable to pathologists. On the other end of the scale, the authors predicted physical labor to harbor a 78% automation potential. The study also emphasized that the automation potential was only one factor to influence human replacement through automation. The other factors were: costs to automate, relative scarcity and costs of skilled replacement workers, benefits beyond labor replacement (quality gains), and lastly, regulatory and social-acceptance considerations. Therefore, technical advances, pathology staff shortage, and quality improvements through DP would have to be regarded as the main drivers for automation pressures on the profession. That said, professions applying medical expertise were still rated as low-risk for automation overall.

Will AI make the pathologist’s workday less taxing? In conjunction with AI, DP may assist with tasks strenuous for human assessment (e.g., quantification). Conversely, AI might more generally be good at what is comparably simple for human evaluation, such as detecting metastases in lymph nodes and might fail where things become difficult for both humans and AI, i.e., complex and challenging evaluations, where overall judgment is needed. This situation could lead to pathologists being left with an assortment of difficult tasks when AI performs the simple ones. We expect AI in DP to result in the pathologist’s future work-day becoming more demanding but also even more interesting.

Even when automation is advanced, the verification of overall plausibility, the quality control, and the medico-legal responsibility will rest with a human professionalist, as is already the case with highly automated, specialized areas of medicine such as laboratory medicine. Inevitably, DP will create a global market for histopathology services, where language barriers, first of all, delineate competing labor-markets. Salaries for pathologists might come under pressure in high-income markets due to increased competition with lower-wage countries. Conversely, in areas with sometimes dire pathology staff shortages, such as parts of Africa and Asia, outsourcing tasks to AI may be beneficial to alleviate staff shortages.

## 13. Important Open Challenges and How They Could Be Addressed

Regarding the problem of non-transparent AI algorithms, explainable AI, which is a subfield of AI, is dedicated to exposing complex AI models to humans in a systematic and interpretable manner and one of the ways to allow for human scrutiny and ultimately the building of trust in this technique. Consequently, not only classification performance but also explainability will need to be goals in AI development. As a near-term strategy, the use of supervised ML has the advantage of generating AI-classifications along established human categories, which in turn increases AI-acceptance. Adequately powered performance studies should help to allay doubts, as consistent results that correlate with clinical parameters are the ultimate goal.

Occupational health evaluations need to systematically address musculoskeletal as well as ocular injury. This necessitates studies dedicated to the pathologist’s profession (akin to radiology [75]) and the routine inclusion of occupational health surveys into DP implementation studies. Appropriate parameters for evaluation need to be defined (e.g., mean hours screen time worked per day) in collaboration with ophthalmologists. Awareness of CVS and possible measures such as ergonomic screen setup, regular breaks, and accurate refractory correction is necessary. Possible benefits of glasses prescribed specifically for computer use should be evaluated, especially in full-time DP users.

Cost-effectiveness analyses based on standardized criteria, as suggested in [41], will provide more robust and comparable data in the future. Results need to be stratified by type of department (academic/non-academic), the extent of DP implementation, and ideally compared to other players in the field working under comparable reimbursement schemes.

## 14. Conclusions

DP undoubtedly holds great potential for routine histopathology in the near future. DP has shown exciting results for primary histopathological diagnoses, even more so with AI applications now becoming embedded into the digital reporting work-flow. Nevertheless, uncertainties as to the current extent of clinically relevant benefits remain in the face of considerable additional costs upfront and hard to compare hospital and reimbursement settings. Table 1 gives an overview of the possible advantages and disadvantages of DP over conventional optical microscopy that we identify. As much of the current work on DP comes from early adopters, some involved in corresponding hardware and software development, a certain degree of enthusiastic bias in favor of DP is inevitable. The current COVID-19 pandemic has clearly given a boost to the field, so more robust real-world data from larger-scale DP implementations can be expected soon.

## Figures and Tables

**Table 1:** Advantages and disadvantages in digital pathology.

Digital Pathology (DP) Feature	Possible Advantages	Possible Disadvantages
In-house telepathology	○ Quick second opinion ○ Social distancing (COVID-19 pandemic)	○ Second opinion overuse (interrupted work-flows) ○ Decreased interpersonal (face-to-face) communication
Extramural telepathology	○ Service for remote/understaffed areas ○ Specialization through DP in low volume labs ○ Home-office use ○ Healthcare cost reduction through global histopathology market	○ Social isolation in remote telepathology ○ Loss of routine on-site expertise through home office ○ Wage competition through global histopathology market
Consultation telepathology	○ Quick access possible ○ No physical slide transfer ○ Lower threshold for consultation due to shorter turnaround time	○ No tissue block available for additional stains/molecular assays ○ Consulted pathologist unaccustomed to work-up (stains/scanner calibration) at the primary center ○ Compatibility issues due to diverse proprietary DP formats ○ Possible medico-legal implications due to restricted work-up
WSI-general	○ No physical slide distribution ○ No fading of stored slides ○ No irretrievable/lost slides ○ Shorter sign-out time ○ Reduced misidentification of slides due to barcoded slides automatically allocated to the case ○ Easy dynamic workload allocation (e.g., management of backlogged work, redistribution in case of sick leave)	○ Time to evaluable-ready slide increased due to additional scan time ○ Integration into a laboratory information system (LIS) for full efficiency gains needed → possible costs for LIS update ○ Regular calibration required (scanners/displays) ○ Small particles omitted by scan → manual checking for rescan ○ Artifacts (out-of-focus areas, digital stitching artifacts) ○ Increased IT-dependence (IT-downtime) compared to optical microscopy
WSI-reporting/user experience	○ Parallel (side-by-side) viewing, digital slide superposition ○ Shorter sign-out time ○ Quick access to prior slides → less immunohistochemistry ○ Facilitates slide presentation at multidisciplinary tumor board ○ Easy image sharing in clinical communication ○ Computational pathology possible (see below) ○ Occupational health: less neck strain, more flexible posture	○ Slower evaluation compared to optical microscopes ○ Mostly only single focus plane in routine DP → difficulties with interpretation ○ Some structures harder to recognize on WSI → glass slide needed ○ Polarization not possible on DP → glass slide needed ○ Extra training for safe practice required (perceived insecurity on digital sign-out) if not DP from career start ○ Easy availability of prior digital slides might shift medico-legal onus towards more extensive re-examination → increased workload ○ Dual infrastructure generally necessary (glass and digital) ○ Occupational health: Computer Vision Syndrome (CVS)
WSI-Image Analysis, ML/AI	○ Faster/efficient and more accurate measurements/quantifications ○ Exact quantification of tumor cell content for molecular analyses ○ Digital enhancement of image features ○ AI for second-read safety net ○ Direct link morphology to clinical parameters “novel biomarker” beyond human recognition ○ Inspection/correction of suggestions from AI-apps in development on WSI-viewer: “human-in-the-loop” interaction	○ Benefit of more accurate quantification not necessarily clinically relevant ○ Applications beyond human evaluation not yet approved/used for clinical management ○ AI intransparent (“black box”) ○ Regulatory oversight challenges with self-modifying (adaptive) AI as algorithm/performance not constant over time
WSI-teaching	○ Digital images for presentation and exams readily available ○ Remote teaching and self-study ○ Increased student motivation, modern appeal	○ None
Costs and efficiency gains	○ Work time saved through faster turnaround times ○ Decreased auxiliary techniques (less immunohistochemistry) ○ Decreased physical slide-transfer costs	○ DP implementation and maintenance and storage costs add to current fixed costs if productivity gains remain unrealized (fixed work contracts) ○ Dual infrastructure costs (workstations and microscopes if kept) ○ Glass and digital storage still generally deemed necessary ○ Technical expert knowledge for hardware acquisitions needed

WSI: whole slide imaging, AI: artificial intelligence, ML: machine learning. Lines are intended as separators between different “digital pathology features“.
